# Supplementary material for: Distribution of pediatric intensive care beds in Brazil: Regional inequalities and implications for equity in the public health system
Source: PLoS One. 2025 Dec 18;20(12):e0339186. doi: 10.1371/journal.pone.0339186 (PMC12714271; doi:10.1371/journal.pone.0339186)
Supplement: Attachment — Submitted filename: REBUTTAL LETTER.docx [file pone.0339186.s003.docx]

**REBUTTAL LETTER – PLOS ONE**

**Response to Reviewers**

**Manuscript title:** *Distribution of Pediatric Intensive Care Beds in Brazil: Regional Inequalities and Implications for Equity in the Public Health System*

Dear Dr. Eric Sribnick,

We thank you, the Academic Editor, and both reviewers for the thoughtful, detailed, and constructive feedback. We appreciate the opportunity to revise our manuscript and believe that the revisions have substantially strengthened clarity, methodological transparency, and analytical rigor.

Below, we provide a **point-by-point response to every comment**, indicating exactly how and where each issue was addressed. Line numbers refer to the revised tracked-changes manuscript.

**Response regarding Data Availability**

We confirm that all raw data required to fully replicate the results of our study are already included in the submission. The Supporting Information files contain the complete minimal dataset used to generate all analyses, tables, and figures, including:

- PICU bed counts by municipality and health region
- Bed typology (Types I, II, III)
- Population denominators (total and <14 years)
- Mortality counts (<14 years)
- Derived variables used in clustering (after standardization)
- Metadata describing variable definitions and data sources
- Full analytic code or steps used to preprocess and merge CNES, SIM, and IBGE data (as applicable)

No restrictions apply to the data. All datasets are derived from public, open-access repositories (DATASUS and IBGE) and have been provided in processed form in the Supporting Information to allow full reproducibility.

We therefore confirm that the submission contains the complete **minimal data set**, as required by PLOS ONE.

**EDITOR COMMENTS**

**Editor comment:**

*“In the discussion, please remove the second mention of how causality cannot be addressed ‘Although causality cannot be confirmed…’ You are correct; however, mentioning this twice is redundant.”*

**Response:**

Thank you for this clarification. We removed the duplicated sentence referencing the inability to infer causality. The discussion now includes only a single, appropriately placed cautionary statement (Revised manuscript, Discussion section).

**REVIEWER #1 COMMENTS AND RESPONSES**

Below, we address each sub-point in sequence:

**1. Comment:**

*“The abstract describes the top-level results… For scientific purposes it should include a brief rationale, study design, objective, source and volume of data, time frame, variables, basis for defining clusters, and more specific recommendations.”*

**Response:**

Rewritten the abstract to include:

- explicit rationale,
- design (“cross-sectional ecological study”),
- data sources (CNES, IBGE, SIM),
- coverage (all 5,570 municipalities and 450 health regions),
- reference month (July 2023),
- variables used (PICU beds by type, mortality, age structure),
- cluster analysis basis (standardization + BIC and silhouette).

(See revised Abstract, lines 56-81.)

**2. Comment:**

*“The introduction can be abridged, moving references to similar studies to the discussion.”*

**Response:**

We shortened the Introduction and moved background examples to the Discussion. (Revised manuscript, Introduction.)

**3. Comment:**

*“Provide a brief description of the source of data (Brazilian databases) for unfamiliar readers.”*

**Response:**

Added concise explanations of CNES, DATASUS, IBGE, and SIM in both Introduction and Methods. (Methods: Design, Population, and Location section.)

**4. Comment:**

*“Consider visual aids: a map of Brazil showing cluster regions.”*

**Response:**

A GIS map was prepared and included as *Figure 1*, showing PICU beds and cluster distribution across health regions. We also mentioned this in the text (lines 310-319)

**5. Comment:**

*“Provide details on cluster analysis preprocessing (e.g., normalization), values, and metrics.”*

**Response:**

We expanded the Methods section to describe:

- variable standardization (z-scores),
- hierarchical clustering algorithm,
- linkage method,
- internal validity metrics (BIC, silhouette).

**6. Comment:**

*“Include a footnote on tables to explain how the clusters were defined.”*

**Response:**

Footnotes added to *Table 3* specifying clustering procedure and criteria.

**7. Comment:**

*“In some regions, mortality rates appear high relative to their PICU distribution. Consider adding confounding factors.”*

**Response:**

We expanded the Discussion with a new subsection addressing confounding influences (HDI, access to emergency care, transport, comorbidities, neonatal care, socioeconomic gradients).

**8. Comment:**

*“Review language for consistency, clarity, long sentences.”*

**Response:**

We revised the manuscript extensively for clarity, concise grammar, and consistent terminology.

**9. Comment:**

*“Discuss limitations: data quality, completeness, confounders.”*

**Response:**

Limitations section expanded to address underreporting, misclassification risk, CNES reliability, and confounders. Cross-referenced relevant literature.

**REVIEWER #2 COMMENTS AND RESPONSES**

**Major Comments**

**1. Comment:**

*“Paper reads as if higher Type III concentration is associated with lower mortality. Clarify correlation vs. causation; consider contextual factors (HDI, poverty, urbanicity…). I urge GIS mapping.”*

**Response:**

We appreciate the reviewer’s observation. In response, we have:

1. Removed or revised any language that could suggest a causal relationship between PICU bed distribution and mortality outcomes.
2. Added explicit statements clarifying that:
   - the study is ecological in design,
   - findings reflect associations rather than causation,
   - results are likely influenced by substantial confounding and context-specific factors.
3. Incorporated a GIS-based map illustrating the national spatial distribution of PICU clusters, now included as Figure 1 to strengthen the geographic interpretation of results.

**2. Comment:**

*“Expand more on PICU Types I, II, III. Are these based on location? Equivalent to AAP Levels I–III? Define monitoring levels.”*

**Response:**

We rewrote the PICU typology section to clarify:

- definitions from ANVISA RDC 7/2010,
- linkage to AAP Level I–III structure,
- typical locations (district vs. tertiary centers),
- infrastructure and monitoring capabilities for each type.

(This can be found in sections “Methods” and “Introduction”.)

**3. Comment:**

*“Mortality rate formula: authors used crude rate. Please compute age-specific rate per 100,000 children <14 and re-run analyses.”*

**Response:**

We recalculated mortality as:

Deaths <14 / Population <14 × 100,000

Updates made to:

- Methods (mortality definition),
- Results tables (recomputed values),
- Cluster analysis (rerun with new indicator),
- Discussion (interpretation adjusted).

This is now corrected throughout.

**4. Comment:**

*“Clarify clustering model (k-means? hierarchical? model-based?). If BIC/AIC used, explain assumptions; if k-means, BIC/AIC not standard.”*

**Response:**

We clarified that we used **hierarchical clustering with Ward’s linkage** on **z-score-standardized variables**.

- BIC was used for model fit through internal cluster evaluation, not model-based Gaussian mixtures.
- Silhouette coefficient reported as main internal validation metric.

**5. Comment:**

*“259 regions have no beds. CNES under-reporting is known; add sensitivity analysis.”*

**Response:**

We added a sensitivity analysis in the Methods (Context section):

- All months of 2023 assessed; variation <1%.
- Discussed CNES underreporting risk in Limitations.
- Cross-checking with SIH-SUS flagged as future direction due to access constraints.

**Minor Comments**

**1. Comment:**

*“July vs. June 2023 discrepancy. Reconcile month choice.”*

**Response:**

We harmonized all references to **July 2023** and justified selection due to seasonal peak.

**2. Comment:**

*“Define mortality period: 2023? multi-year?”*

**Response:**

Clarified: **mortality refers to calendar year 2023** (SIM data).

**3. Comment:**

*“Confirm feature scaling.”*

**Response:**

Confirmed and added: **z-score standardization** was applied.

**4. Comment:**

*“Explain denominator: population appears 173M vs. Brazil’s 203M.”*

**Response:**

Thank you for identifying this issue. We reviewed the population figures and confirmed that the correct total population for Brazil, based on the 2022 IBGE estimates, is approximately 203 million. We corrected this value in the manuscript to ensure consistency with official national statistics.

Because the discrepancy was due only to a numerical error rather than a methodological distinction, no additional explanation was required in the Methods section.

The corrected population value now appears throughout the revised manuscript and in all affected tables.

**5. Comment:**

*“Using CNES Type I/II/III labels—provide validation comment or misclassification risk.”*

**Response:**

Added a paragraph discussing CNES classification reliability, referencing RDC 7/2010 and literature on CNES data quality (Rocha et al. 2018; Lima et al. 2009).

**6. Comment:**

*“Include a proxy for access (distance/time to nearest PICU III).”*

**Response:**

Thank you for this important suggestion. We agree that geospatial access indicators—such as distance or travel time to the nearest Type III PICU—are highly relevant to understanding regional disparities in critical care. However, several considerations prevented us from incorporating these metrics into the present study.

First, this specific analysis is the subject of a planned future study, in which we intend to apply detailed geospatial methods (e.g., road-network routing, travel-time modelling, and population-weighted centroids).

Second, in the current manuscript, our objective was not to evaluate geographic access, network organization, or health-planning logistics, nor to construct a conceptual framework for the configuration of pediatric critical care networks. Instead, our focus was strictly on describing the distribution and technological complexity of PICU beds and examining broad ecological associations with pediatric mortality.

Third, Brazil’s geographic and administrative structure differs significantly from countries where centroid-based modeling is routinely used. In Brazil, the “centroid” of a health region is not a geographic midpoint but rather a politically defined administrative reference, which may not correspond to population distribution or actual service locations. Using this administrative centroid as a proxy for geographic access would therefore introduce systematic bias and potentially distort estimates of distance or travel time.

For these reasons, incorporating access proxies would require a dedicated methodological framework beyond the scope of this ecological study. Nevertheless, we have added language in the Discussion acknowledging this limitation and outlining the importance of future geospatial analyses.

We also included a GIS-based illustrative map (Figure 1) showing the spatial distribution of PICU beds and clusters, which offers additional geographical context.

**7. Language comments (abstract, terminology, acronyms).**

**Response:**

All requested corrections made:

- “adolescentes” corrected to “adolescents”.
- Month/year and units added.
- Consistent terminology (health regions vs. macro-regions).
- Defined all acronyms on first use.

**CONCLUSION**

We thank the Academic Editor and both reviewers for their exceptionally thorough and constructive feedback. We believe the revised manuscript is now substantially stronger—methodologically clearer, more transparent, and more cautious in interpretation while also incorporating new analyses and clarifications.

We remain at your disposal for any further revisions.

Sincerely,
**Luís Felipe Ribeiro Soares (on behalf of all authors)**
